# Hypoglycemia Assessed by Continuous Glucose Monitoring Is Associated with Preclinical Atherosclerosis in Individuals with Impaired Glucose Tolerance

**DOI:** 10.1371/journal.pone.0028312

**Published:** 2011-12-02

**Authors:** Ersilia Castaldo, Donata Sabato, Davide Lauro, Giorgio Sesti, Maria Adelaide Marini

**Affiliations:** 1 Department of Internal Medicine, University of Rome-Tor Vergata, Rome, Italy; 2 Department of Experimental and Clinical Medicine, University Magna Græcia of Catanzaro, Catanzaro, Italy; University of Padova, Italy

## Abstract

Hypoglycemia is associated with increased risk of cardiovascular adverse clinical outcomes. There is evidence that impaired glucose tolerance (IGT) is associated with cardiovascular morbidity and mortality. Whether IGT individuals have asymptomatic hypoglycemia under real-life conditions that are related to early atherosclerosis is unknown. To this aim, we measured episodes of hypoglycemia during continuous interstitial glucose monitoring (CGM) and evaluated their relationship with early manifestation of vascular atherosclerosis in glucose tolerant and intolerant individuals. An oral glucose tolerance test (OGTT) was performed in 79 non-diabetic subjects. Each individual underwent continuous glucose monitoring for 72 h. Cardiovascular risk factors and ultrasound measurement of carotid intima-media thickness (IMT) were evaluated. IGT individuals had a worse cardiovascular risk profile, including higher IMT, and spent significantly more time in hypoglycemia than glucose-tolerant individuals. IMT was significantly correlated with systolic (*r* = 0.22; *P* = 0.05) and diastolic blood pressure (*r* = 0.28; *P* = 0.01), total (*r* = 0.26; *P* = 0.02) and LDL cholesterol (*r* = 0.27; *P* = 0.01), 2-h glucose (*r* = 0.39; *P*<0.0001), insulin sensitivity (*r* = −0.26; *P* = 0.03), and minutes spent in hypoglycemia (*r* = 0.45; *P*<0.0001). In univariate analyses adjusted for gender, minutes spent in hypoglycemia were significantly correlated with age (*r* = 0.26; *P* = 0.01), waist circumference (*r* = 0.33; *P* = 0.003), 2-h glucose (*r* = 0.58; *P*<0.0001), and 2-h insulin (*r* = 0.27; *P* = 0.02). In a stepwise multivariate regression analysis, the variables significantly associated with IMT were minutes spent in hypoglycemia (r^2^ = 0.252; *P*<0.0001), and ISI index (r^2^ = 0.089; *P* = 0.004), accounting for 34.1% of the variation. Episodes of hypoglycemia may be considered as a new potential cardiovascular risk factor for IGT individuals.

## Introduction

Hypoglycemia is a well-known side effect of glucose-lowering therapy in both type 1 and type 2 diabetes mellitus, and becomes more prevalent with treatment intensification [1], [2]. Hypoglycemia has been strongly associated with increased risk of cardiovascular adverse clinical outcomes [1]–[4]. Hypoglycemia may promote localized vasoconstriction by inducing release of epinephrine, it may also induce pro-inflammatory, platelet aggregatory, anti-fibrinolytic, and pro-thrombotic changes, and stimulate stress responses leading to endothelial damage, and vascular atherosclerosis [5], [6]. There is evidence that impaired glucose tolerance (IGT) is associated with cardiovascular morbidity and mortality [7]–[9]. Subjects with IGT are characterized by a late insulin response to an oral glucose tolerance test (OGTT), that results in a progressive raise of plasma insulin from 60 to 120 min [10], [11]. In these subjects, a persistent increase in insulin levels after a meal may result in late postprandial hypoglycemia. Whether IGT individuals have asymptomatic hypoglycemia under real-life conditions that are related to early atherosclerosis is unknown. To this aim, we measured episodes of hypoglycemia during continuous interstitial glucose monitoring (CGM) and evaluated their relationship with a early manifestation of vascular atherosclerosis, assessed as carotid intima–media thickness (IMT) in glucose tolerant and intolerant individuals.

## Materials and Methods

Seventy-nine non-diabetic Caucasian subjects were consecutively recruited at the Department of Internal Medicine of the University of Rome-Tor Vergata. Recruitment mechanisms include word-of-mouth, fliers, and newspaper advertisements. Subjects, aged 23–70 years, were excluded if they had history of cardiovascular disease, including peripheral atherosclerosis, chronic gastrointestinal diseases associated with malabsorption, chronic pancreatitis, history of any malignant disease, history of alcohol or drug abuse, liver or kidney failure, or received treatments able to modify glucose metabolism including glucose-lowering, lipid-lowering and antihypertensive therapy. None of the subjects were taking anti-platelet medications. All subjects underwent an anthropometrical evaluation and readings of clinic blood pressure were obtained in the left arm of the supine patients, after 5 min of quiet rest, with a mercury sphygmomanometer. Systolic blood pressure (SBP) and diastolic blood pressure (DBP) were recorded at the first appearance (phase I) and at the disappearance (phase V) of Korotkoff sounds. Blood pressure values were calculated as the average of the last two of three consecutive measurements obtained at 3-mins intervals. After 12-h fasting, a 75 g OGTT was performed with 0, 30, 60, 90 and 120 min sampling for plasma glucose and insulin. The Matsuda index of insulin sensitivity (ISI) was calculated as reported [12]. Each individual underwent CGM for 72 h with a Medtronic MiniMed, CA, a Holter-style sensor system designed to continuously monitor interstitial fluid glucose levels within a range of 40–400 mg/dl. The sensor was inserted in the subcutaneous abdominal fat tissue in accordance with the manufacturers' instructions. Initial sensor failure was defined as unsuccessful calibration on repeated attempst over 1 hour, current and voltage signals fluctuating rapidly or out of range, or haemorrhage at the insertion site, when the sensor was removed and replaced with a new sensor at a different site. Subjects were taught calibration procedures and instructed to calibrate whenever alerted by the device. According to the accuracy criteria defined by the manufacturer, in our study CGM results were considered valid if at least three calibrations were performed during 24-h of monitoring. Sensor insertions were performed in hospital, but subjects were dismissed, and encouraged to maintain their usual daily diet and activities during the monitoring period. CGM data were analyzed using Medtronic-MiniMed Solutions Software version 3.0b. As an indicator of hypoglycemia, the time spent below 70 mg/dl blood glucose [13] were calculated during the 72 h glucose monitoring period.

IMT of the common carotid artery was measured by ATL HDI 3000 ultrasound system (Advanced Technology Laboratories, Bothell, WA) equipped with a 5 MHz linear array transducer as previously described [14], [15]. This system provides high resolution ultrasonic images with 0.3 mm axial resolution. The subjects were examined in the supine position. To avoid variability during the cardiac cycle, the images were frozen in the end-diastolic phase. IMT was determined only from the far wall of the artery, because it is known to have a higher precision than the near arterial wall. Manual measurements were conducted in plaque-free portions of the 10-mm linear segment proximal to the carotid bulb. For each patient two measurements were performed bilaterally, and the values were averaged, which presented the mean of IMT of the common carotid artery. No plaques, defined as a clearly isolated focal thickening of the intima-media layer with a thickness >1.3 mm, were observed in any individuals. Ultrasound study was performed by an experienced examiner who was unaware of the subjects' clinical and laboratory findings.

The protocol was approved by the institutional ethical committee the University of Rome-Tor Vergata and informed written consent was obtained from each participant in accordance with principles of the Declaration of Helsinki.

### Statistical analysis

Continuous data are expressed as means ±SD. Categorical variables were compared by χ^2^ test. The coefficient of variation (CV) of interstitial glucose during CGM was calculated as the ratio of the standard deviation to the mean. Phenotypic differences between groups were tested after adjusting for age, and gender using a general linear model. Partial correlation coefficients adjusted for age and gender were computed between variables. Relationships between variables were sought by stepwise multivariate linear regression analysis to assess their independent contribution to IMT. A *P* value<0.05 was considered statistically significant. All analyses were performed using SPSS software Version 16.0.

## Results

**Figure 1:**
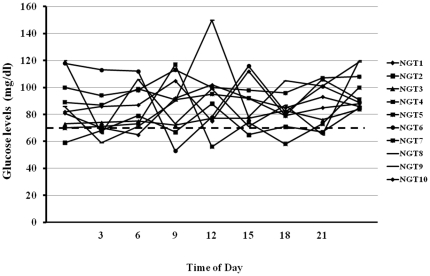
Twenty-four-hour glucose profiles of 10 representative individuals with NGT. Dashed line indicates the 70 mg/dl glucose threshold.

**Figure 2:**
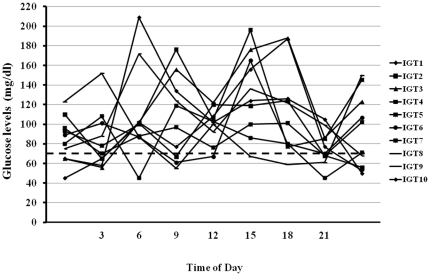
Twenty-four-hour glucose profiles of 10 representative individuals with IGT. Dashed line indicates the 70 mg/dl glucose threshold.

The anthropometric and cardio-metabolic characteristics of the study groups are shown in Table 1. IGT individuals were older, more frequently smokers, and had significantly higher lipid levels, blood pressure, 2-hour post-load plasma glucose and insulin levels, and carotid IMT, as well as lower insulin sensitivity, assessed by the ISI index, as compared with glucose tolerant individuals (Table 1). IGT individuals spent significantly more time in hypoglycemia as compared with glucose tolerant individuals (Table 1 and Figures 1 and 2). Mean interstitial glucose levels during CGM did not differ between the two groups of subjects. CV of interstitial glucose during CGM was not significantly higher in IGT individuals. In univariate analyses adjusted for gender, minutes spent in hypoglycemia were significantly correlated with age (*r* = 0.26; *P* = 0.01), waist circumference (*r* = 0.33; *P* = 0.003), 2-h post-load plasma glucose (*r* = 0.58; *P*<0.0001), and 2-h post-load insulin (*r* = 0.27; *P* = 0.02). In univariate analyses adjusted for gender and age, carotid IMT was significantly correlated with systolic and diastolic blood pressure, total and LDL cholesterol, 2-h post-load plasma glucose, insulin sensitivity, and minutes spent in hypoglycemia (Table 2). A stepwise multivariate regression analysis in a model including age, gender, body mass index, waist circumference, smoking habits, lipid levels, blood pressure, fasting and 2-hour post-load plasma glucose, fasting and 2-hour post-load insulin levels, ISI index, and minutes spent in hypoglycemia showed that the two variables that remained significantly associated with carotid IMT were minutes spent in hypoglycemia (partial r^2^ = 0.252; *P*<0.0001), and ISI index (partial r^2^ = 0.089; *P* = 0.004), accounting for 34.1% of the variation.

**Table 1:** Anthropometric and biochemical characteristics of the study subjects.

	NGT subjects	IGT subjects	*P*
Number (*male/female*)	36 (16/20)	43 (27/16)	NS
Age *(yrs)*	50±11 (27–70)	58±9 (23–70)	<0.01*
BMI *(kg/m^2^)*	27.4±5.7 (20.3–41.7)	27.0±3.5 (19.9–34.3)	NS
Waist circumference (*mm*)	90±17 (62–140)	96±13 (65–129)	NS
Smokers (%)	12 (33.3%)	24 (55.8%)	<0.05
SBP *(mmHg)*	125±17 (95–170)	135±16 (100–180)	<0.05
DBP *(mmHg)*	77±12 (55–100)	83±8 (65–100)	<0.05
Total Cholesterol *(mg/dl)*	191±37 (110–270)	207±28 (142–271)	<0.05
LDL Cholesterol *(mg/dl)*	118±37 (22–200)	133±27 (77–197)	<0.05
HDL Cholesterol *(mg/dl)*	48±11 (27–69)	43±11 (20–69)	<0.05
Triglyceride *(mg/dl)*	123±42 (49–190)	152±50 (52–273)	<0.05
Fasting Glucose *(mgl/dl)*	104±9 (79–124)	105±11 (83–125)	NS
2-h glucose (*mg/dl*)	108±18 (65–139)	176±19 (144–199)	<0.0001
Fasting Insulin *(µU/ml)*	13±7 (3–37)	12±7 (1–25)	NS
2-h Insulin *(µU/ml)*	67±57 (19–308)	102±81 (27–451)	<0.05
Matsuda index/ISI	5.2±3.5 (1.9–18.7)	3.3±1.2 (0.9–6.3)	<0.05
Intima-media thickness (*mm*)	0.73±0.09 (0.6–0.9)	0.87±0.13 (0.45–1.0)	<0.0001
Minutes per day in hypoglycemia	35±12 (5–59)	130±74 (5–250)	<0.0001
Mean interstitial glucose during CGM	98±22 (74–137)	109±26 (74–234)	NS
Coefficient of variation (CV) of interstitial glucose during CGM	22 (7–61) (95% CI 25 to 32)	28 (10–58) (95% CI 24 to 31)	

Data are means ± SD. Data in brackets are ranges of minimum and maximum value. Data of CV of interstitial glucose during CGM are reported as the mean, the range and the 95% CI for comparison between the groups. Categorical variables were compared by χ2 test. *P* values refer to results after analyses with adjustment for age, and gender using a general linear model.

*P values refer to results after analysis with adjustment for gender using a general linear model. BMI: body mass index; SBP = systolic blood pressure; DBP = diastolic blood pressure. NS = Not significant.

**Table 2:** Univariate correlations between IMT and anthropometric and biochemical variables.

	IMT	*P*
	*r*	*P*
Age *(yrs)*	0.29	<0.01*
BMI *(kg/m^2^)*	0.13	NS
Waist circumference (*mm*)	0.21	NS
SBP *(mmHg)*	0.22	<0.05
DBP *(mmHg)*	0.28	<0.01
Total Cholesterol *(mg/dl)*	0.26	<0.05
LDL Cholesterol *(mg/dl)*	0.27	<0.01
HDL Cholesterol *(mg/dl)*	−0.08	NS
Triglyceride *(mg/dl)*	0.07	NS
Fasting Glucose *(mgl/dL)*	0.03	NS
2-h glucose (*mg/dl*)	0.39	<0.0001
Fasting Insulin *(µU/ml)*	0.18	NS
2-h Insulin *(µU/ml)*	0.13	NS
Matsuda index/ISI	−0.26	<0.05
Minutes per day in hypoglycemia (<70 mg/dl)	0.45	<0.0001
Mean interstitial glucose during CGM	0.12	NS
Coefficient of variation (CV) of interstitial glucose during CGM	0.08	NS

Partial correlation coefficients adjusted for age and gender were computed between variables. *P* values refer to results after analyses with adjustment for age, and gender.

*P values refer to results after analysis with adjustment for gender. BMI = Body Mass Index, SBP = systolic blood pressure; DBP = diastolic blood pressure.

## Discussion

To the best of our knowledge, this is the first study evaluating the association between episodes of hypoglycemia, assessed by CGM under real-life conditions, and preclinical atherosclerosis in non-diabetic subjects with different degrees of glucose tolerance. Our findings suggest that IGT individuals have asymptomatic episodes of hypoglycemia and spend more than 2 hrs per day below the hypoglycemia threshold of <70 mg/dl. The hypoglycemic episodes were significantly correlated with 2-h post-load plasma glucose and insulin levels, thus raising the possibility that hypoglycemia occurs during the late post-prandial period as a consequence of a prolonged release of insulin in response to elevated glucose levels.

Performance assessment of the Medtronic-MiniMed CGM system has demonstrated that it has an acceptable clinical accuracy, with 96.6% of paired sensor-blood glucose self monitoring readings falling in the ‘clinically acceptable’ zones A and B of the Clarke error grid for type 1 diabetic subjects [16]. As compared with blood glucose self monitoring, CGMS detected significantly more episodes of hypoglycemia and post-prandial hyperglycemia, while total duration of hyperglycemia, blood glucose oscillations and day-to-day variability were assessed with a similar accuracy with the two methods [16]. Additionally, no statistically significant differences between Medtronic-MiniMed CGM system measures and self-monitoring capillary plasma glucose readings have been observed in non-diabetic individuals [17].

The measurement of IMT of the common carotid artery is a well accepted method to monitor the early stages of atherosclerosis and IMT increase precedes the development of plaque and stenosis in the arterial wall. Furthermore, IMT of the common carotid artery has been shown to be related to prevalent and incident cardiovascular disease [18]. There is incomplete information regarding determinants of vascular damage in IGT. We found that the time spent in hypoglycemia was the strongest determinant of carotid IMT. Alterations in vascular tone, coagulation, fibrinolysis, and inflammation associated with repeated episodes of hypoglycemia may be associated with the induction and progression of atherosclerosis [5], [6]. These findings suggest that the number of asymptomatic episodes of hypoglycemia could be considered as a new potential cardiovascular risk factor in IGT individuals. However, in the absence of data determining the impact of these episodes of hypoglycemia on major cardiovascular outcomes, the present results cannot establish whether episodes of hypoglycemia are part of normal variability in IGT individuals or whether such episodes affect the risk of progression to cardiovascular disease.

Although observational studies have reported a strong association between hyperglycemia and increased risk for cardiovascular disease in individuals with type 2 diabetes, the results of recent interventional randomized controlled trials in establishing the benefit of intensive glycemic control on cardiovascular outcomes have been elusive [19]–[22]. The glycemic control arm of the Action to Control Cardiovascular Risk in Diabetes (ACCORD) was halted early because of higher mortality in the intensive arm compared with the standard arm [20]. The underlying reason of these findings was unclear at the time of the intervention stop, although several hypotheses were raised, including severe hypoglycemia. In a post hoc analysis of the ACCORD study, it was found that patients who experienced severe hypoglycemia, regardless of study arm, showed an increased risk of death [23]. Accordingly, in the Action in Diabetes and Vascular Disease: Preterax and Diamicron Modified Release Controlled Evaluation (ADVANCE) study, severe hypoglycemia was associated with increased risk of cardiovascular events, and death from both cardiovascular and non-cardiovascular causes in patients assigned to either standard or intensive glucose control [24]. The detrimental role of hypoglycemia is further supported by a retrospective cohort study including data from the UK General Practice Research Database showing that both low and high mean HbA1c values were associated with increased all-cause mortality according to an U-shaped pattern of risk [25].

This study has some strengths including the measurement of plasma glucose during an OGTT in drug-naïve individuals thus excluding the confounding effect of medications affecting glucose, and assessment of CGM under real-life conditions. Nonetheless, the present study has limitations. CGM has intrinsic limitations since it measures interstitial fluid rather than blood glucose levels. In addition, OGTTs were performed once, and, thus, intra-individual variation in plasma glucose levels cannot be taken into account leading to possible misclassification of some subjects with IGT. The cross-sectional design of the study does not provide insights into the time course of the development of cardiovascular disease, and, therefore, no conclusions regarding cause-effect relationships can be made. Finally, the present findings are only based on Caucasian individuals, and results might vary as a function of ethnic group.

In conclusion, since hypoglycemic episodes are considered to be among the mediators of cardiovascular events, our findings indicate that exposure to glucose levels below the hypoglycemia threshold remains under-appreciated in individuals with IGT. The present results suggest that it is common for persons who are classified as ‘pre-diabetic” to experience transient hypoglycemia during everyday circumstances.
